# The family experiences of in-hospital care questionnaire in severe traumatic brain injury (FECQ-TBI): a validation study

**DOI:** 10.1186/s12913-016-1884-6

**Published:** 2016-11-28

**Authors:** Audny Anke, Unn Sollid Manskow, Oddgeir Friborg, Cecilie Røe, Cathrine Arntzen

**Affiliations:** 1 Department of Rehabilitation, University Hospital of North Norway, Sykehusvn.1, 9038 Tromsø, Norway; 2 Faculty of Health Sciences, Department of Clinical Medicine, UiT The Arctic University of Norway, Tromsø, Norway; 3 Faculty of Health Sciences, Department of Health and Care Sciences, UiT The Arctic University of Norway, Tromsø, Norway; 4 Faculty of Health Sciences, Department of Psychology, UiT The Arctic University of Norway, Tromsø, Norway; 5 Department of Physical Medicine and Rehabilitation, Oslo University Hospital, Oslo, Norway; 6 Institute of Health and Society, Research Centre for Habilitation and Rehabilitation Models and Services (CHARM), Faculty of Medicine, University of Oslo, Oslo, Norway

**Keywords:** Parent satisfaction, Patient satisfaction, Family satisfaction, Quality of care, Traumatic brain injury, Rehabilitation

## Abstract

**Background:**

Family members are important for support and care of their close relative after severe traumas, and their experiences are vital health care quality indicators. The objective was to describe the development of the Family Experiences of in-hospital Care Questionnaire for family members of patients with severe Traumatic Brain Injury (FECQ-TBI), and to evaluate its psychometric properties and validity.

**Methods:**

The design of the study is a Norwegian multicentre study inviting 171 family members. The questionnaire developmental process included a literature review, use of an existing instrument (the parent experience of paediatric care questionnaire), focus group with close family members, as well as expert group judgments. Items asking for family care experiences related to acute wards and rehabilitation were included. Several items of the paediatric care questionnaire were removed or the wording of the items was changed to comply with the present purpose. Questions covering experiences with the inpatient rehabilitation period, the discharge phase, the family experiences with hospital facilities, the transfer between departments and the economic needs of the family were added. The developed questionnaire was mailed to the participants. Exploratory factor analyses were used to examine scale structure, in addition to screening for data quality, and analyses of internal consistency and validity.

**Results:**

The questionnaire was returned by 122 (71%) of family members. Principal component analysis extracted six dimensions (eigenvalues > 1.0): acute organization and information (10 items), rehabilitation organization (13 items), rehabilitation information (6 items), discharge (4 items), hospital facilities-patients (4 items) and hospital facilities-family (2 items). Items related to the acute phase were comparable to items in the two dimensions of rehabilitation: organization and information. All six subscales had high Cronbach’s alpha coefficients >0.80. The construct validity was confirmed.

**Conclusion:**

The FECQ-TBI assesses important aspects of in-hospital care in the acute and rehabilitation phases, as seen from a family perspective. The psychometric properties and the construct validity of the questionnaire were good, hence supporting the use of the FECQ-TBI to assess quality of care in rehabilitation departments.

**Electronic supplementary material:**

The online version of this article (doi:10.1186/s12913-016-1884-6) contains supplementary material, which is available to authorized users.

## Background

The family perspective in health care after injuries is recognized as increasingly important and a vital health care indicator [1]. In patients with memory and communication problems experiences of the family members are key dimensions of health care quality, as relatives play a role in care and support and often act as the patient’s representative [2]. After traumatic brain injuries spouses and parents are the most frequent caregivers [3, 4] and may have specific post-trauma experiences and needs [4–6] that change across the treatment phases [7–9]. However, multidimensional scales evaluating care experiences and satisfaction with acute care and rehabilitation after traumatic brain injuries in adult caregivers are not available.

Patients’ experiences and satisfaction with health care services are linked to important aspects of quality of care such as patient adherence to treatment, patient safety and clinical effectiveness [10, 11]. Several national surveys in Norway have described the systematic development and validation of questionnaires related to patients’ experiences [12–14]. The concepts of satisfaction and experiences are positively related and often used interchangeably [14]. Asking patients about their specific experience with concrete events is more valid and easier to interpret than satisfaction ratings [13, 15, 16]. The evidence-based knowledge of patients’ treatment experiences following severe traumatic brain injury (TBI) is limited [17, 18] Important areas for quality of care include information from staff and the organization of services. Furthermore, studies using qualitative methodologies have identified interdepartmental transitions between acute care and inpatient rehabilitation, and the discharge period from hospital, as particularly challenging factors [7, 19].

There are family satisfaction questionnaires for use in the intensive care units [2, 20, 21], and parents’ views have been increasingly used in the evaluation of care quality for children [22, 23]. Theoretically close relatives to surviving injured patients have comparable in-hospital experiences to parents with chronically ill children, so we searched for a suitable parent experience instrument covering multiple dimensions of care. Based on a literature review, the carefully constructed and validated multidimensional parent experience of paediatric care (PEPC) questionnaire [22] represent a suitable starting point for the present development of a questionnaire evaluating family experiences of care after TBI. An intention, not covered in the PEPC questionnaire, was to evaluate separately the acute care and the inpatient rehabilitation phases, and to use a multidisciplinary approach with information about experiences with “staff” rather than “nursing” and “doctor services”. Moreover the development of questionnaires for new groups must incorporate their specific experiences, which focus group interviews may provide [13].

Accordingly, the main aim of this study was to describe the developmental process and to psychometrically evaluate a questionnaire that assesses treatment and rehabilitation experiences of the family members of patients with severe TBI. An intention was to record quality of care experiences separately for different phases of care to be able to make relevant comparisons. The questionnaire was named the family experiences of in-hospital care questionnaire in severe traumatic brain injury (FECQ-TBI), and was evaluated with regard to data quality, factor and scale structure, internal consistency and construct validity.

Based on previous literature on satisfaction with care, including the experiences and satisfaction of relatives (parents), we expected the subscales of the present instrument to be positively and significantly correlated, as experiences with the organization, information and preparation for discharge reflect different but related dimensions of quality of care. Also experiences with hospital facilities were expected to show positive correlations with the other subscales. As all the subscales in the instrument represent aspects of quality of care, positive correlations were hypothesised to exist between subscales and overall satisfaction with care, treatment and rehabilitation. Negative correlations were hypothesised to exist between single-item questions assessing the extent of any incorrect treatment or experiences of problems/disappointments with staff and the subscales regarding experiences with organization and information [22–24]. Finally, as a part of the test of construct validity, the age of the patients was hypothesised to be unrelated to the FECQ-TBI subscales [25].

## Methods

To ensure the content validity in the questionnaire developmental process, items from previous questionnaires assessing the experience of in-hospital patients [12–14] and parents [22, 23] were consulted. Further, selection of items was based on interviews with family members and the expert knowledge of the authors (i.e., UMS, CA and AA) on rehabilitative care after severe TBI or other acquired brain injuries. The psychometric evaluation of the new questionnaire used the COnsensus-based Standards for the selection of health status Measurement Instruments (COSMIN) [26] checklist as a guideline.

### Questionnaire development – the starting point

Following a literature search, the PEPC questionnaire developed by Garratt et al. [22] represented a good starting point for the present FECQ-TBI as it was found to be well validated, not disease specific, and available in Norwegian. The PEPC questionnaire contains 25 questions covering six subscales: organization, information about examinations and tests, doctor services, nursing services, information about discharge and hospital facilities. In addition, included in the PEPC questionnaire was several other single questions of possible interest in the family experiences questionnaire developmental process: nine questions about health care delivery, overall satisfaction with care and how the parents were treated, the extent to which parent expectations were met, information about medication, extent of poor treatment, and extent of any problems with the staff. Good validity, internal consistency and test-retest reliability have been reported [22, 27].

### Focus group

The original PEPC questionnaire was modified for use in the present study after using a focus group approach with three caregivers of patients with severe TBI in northern Norway who had received inpatient rehabilitation. Focus group sizes are usually between 4 and 12 participants per group [28]. The caregivers differed with respect to strategically important aspects such as caregivers’ age and relationship to patient, and patient-related aspects such as type and severity of injury. A focus group is useful for generating in-depth information about the phenomena relevant for the particular study objective [29, 30] such as questionnaire construction. Caregivers’ experiences provide knowledge that through systematic exploration and comparison may contribute to achieve conceptual clarity or identify new conceptual issues [29, 30]. The method is also suitable for examining whether the questionnaire items are appropriately formulated. An interview guide was developed (by authors CA and AA). The interview lasted approximately two hours and was led by a qualified researcher (CA). The interview was audiotaped and one observer (AA) wrote detailed notes throughout the session. The interview included a presentation of the purpose of the interview and an invitation for family members to briefly introduce themselves. The researcher then facilitated a discussion of topics related to treatment phases (acute care, rehabilitation, discharge) using open-ended questions regarding experiences with the staff (e.g., stability, information), good and bad experiences, issues related to safety and trust regarding the treatment quality, areas of high satisfaction, missing health care services, the hospital facilities and preparations for discharge. Lastly, the family members were asked for advice on how to improve the services.

### The new family experiences of care questionnaire in traumatic brain injury (version 1)

Detailed notes from the focus group interview were systematically processed and categorised and the themes that emerged were checked against the items in the PEPC questionnaire. Items that covered important areas were kept in the new questionnaire, often with slight modifications to be suitable. A small pilot study (*n* = 3) of the questionnaire was conducted in which the family members of the focus group commented on the relevance and intelligibility of the questions and evaluated the rated response options on each item. After small changes in wording and the removal of one item, the complete questionnaire yielded 55 items and was named the FECQ-TBI (version 1). Fifty-one items were related to experiences with or satisfaction with care, while four items were yes/no questions asking separately about 1) whether a patient intended diary were used, and 2) whether children were involved, within the acute department and the rehabilitation department, respectively.

Table 1 summarises the 51 care experiences items in the mailed questionnaire (version 1). For each item the corresponding scale in the PEPC questionnaire is given (Table 1, 3rd column, PEPC scale given in table subtext). Some of the questions were modified in the FECQ-TBI; the most frequent modifications of phrases in the FECQ-TBI are the changes from nurse or doctor to staff and from child to patient. For instance, in the PEPC questionnaire, “Nursing staff caring for child” and “Doctors caring for child” are replaced in the FECQ-TBI with “Staff caring for patient”. The 11 questions regarding the acute phase/department were asked with identical phrasing regarding the rehabilitation department. An additional nine items were added regarding experiences with the rehabilitation department as a result of the focus group interview (rehab department items 12–20). New items emerging from the focus group interview were also added to the original PEPC scales discharge-information and hospital facilities. Based on theoretical considerations of distinct concepts, the items relating to discharge and to hospital facilities were analysed as distinct concepts (see Statistics). As illustrated in Table 1, four of the overall single-item questions were identical to the overall questions in the PEPC questionnaire, and three were added in this study: regarding transfer between departments, economical needs and care of involved children. These overall single-item questions were not suitable for factor analysis, as they were never meant to be included in factors or scales [12, 13, 22].

**Table 1:** Overview of the 51 items divided in main areas and illustrating the corresponding questions asked about the acute and rehabilitation departments (version 1 questionnaire)

	Items		Rehab department items			Acute department items		
		PEPC Item^c^	Missing n (%)^b^	Mean^a^	SD	Missing n (%)^b^	Mean^a^	SD
1	One doctor mainly responsible	O	4 (3.5)	3.86	1.11	2 (1.6)	3.39^e^	1.25
2	Fixed group nurses	O	4 (3.5)	3.92	0.98	2 (1.6)	3.85	0.99
3	Staff collaboration	O	4 (3.5)	3.92	0.96	4 (3.3)	4.21	0.83
4	Care/rehabilitation well planned	O	4 (3.5)	3.93	1.00	3 (2.5)	4.19	0.95
5	Thoughtfulness, care for **the patient**	Ns/Ds	3 (2.7)	4.20	0.88	3 (2.5)	4.44	0.71
6	Seemed professionally competent	Ns^d^/Ds	3 (2.7)	4.17	0.94	2 (1.6)	4.49	0.72
7	Information tests, examinations	I	5 (4.4)	3.61^e^	1.10	2 (1.6)	4.09	0.98
8	Took account of family situation	Ns	3 (2.7)	3.79	1.02	2 (1.6)	3.94	1.02
9	Thoughtfulness, care **for relative**	Ns/Ds	4 (3.5)	3.55	1.19	1 (0.9)	3.93	1.03
10	Interested in your opinions	Ns/Ds	3 (2.7)	3.47	1.17	1 (0.9)	3.72	1.15
11	Gave understandable information	Ns/Ds	4 (3.5)	3.89	1.05	1 (0.9)	4.17	0.93
12	Fixed group of other therapists	-	4 (3.5)	4.10	0.93			
13	Felt assure regarding necessary care	-	4 (3.5)	4.02	1.13			
14	Explanation purpose of rehabilitation	-	3 (2.7)	3.67	1.11			
15	Staff committed themselves to patient	-	3 (2.7)	4.05	0.95			
16	Had a fixed contact (rehabilitation)	-	3 (2.7)	3.31	1.15			
17	Provided assistance with the patient	Ns	6 (5.3)	3.90	1.02			
18	Provided coordinated information	-	4 (3.5)	3.73	1.07			
19	Received information about rights	-	3 (2.7)	2.82	1.25			
20	Informed about what you could contribute with at the hospital	-	4 (3.5)	3.16	1.24			
	Discharge period							
1	Information period after discharge	DI	7 (5.7)	3.17	1.30			
2	Felt confident managing follow-up	DI	9 (7.4)	3.24	1.21			
3	Informed about what you could do in the event of problems after discharge	DI	9 (7.4)	2.81	1.38			
4	Informed about short/long term consequences of head injuries	-	6 (4.9)	2.99	1.36			
5	Consulted during planning discharge^e^	-	31 (25.4)	3.51	1.37			
6	Necessary arrangements for further rehabilitation^e^	-	30 (24.6)	3.09	1.26			
	Hospital facilities							
1	Cleanliness	HF	6 (5.3)	4.32	0.85			
2	Bathroom/shower/toilet facilities	HF	5 (4.4)	4.14	0.90			
3	Peace and quiet patient’s room	HF	5 (4.4)	4.38	0.75			
4	Meals for the patient	-	8 (7.0)	4.23	0.93			
5	Rest room/accommodation relative(s)	-	26 (23.0)	3.08	1.44			
6	Meals for the relative(s)	-	22 (19.5)	3.60	1.36			
7	Activity provisions for the patient^e^	HF^d^	8 (7.0)	3.85	1.13			
	Overall single questions about the time in hospital as a whole							
1	Overall satisfaction with care, treatment and rehabilitation at the hospital	PEPC-S	2 (1.6)	4.36	0.82			
2	Overall satisfaction with the way you were treated as a relative	PEPC-S	2 (1.6)	4.23	0.90			
3	Do you believe that the patient in any way received the wrong treatment?^f^	PEPC-S	2 (1.6)	4.43	0.99			
4	Were you angry, distressed, or disappointed with the staff?^f^	PEPC-S	2 (1.6)	3.78	1.32			
5	Any adverse incidents in connection with transfer between departments^f^	-	2 (1.6)	3.68	1.43			
6	Were financial needs taken care off?	-	3 (2.5)	2.29	1.43			
7	Were involved children taken care off (*n* = 53 had children involved)	_	14 (26.4)	3.21	1.28			

^a^Items and scales are scored 1–5 where 5 is the best experience

^b^Percentages of missing items are for the acute phase, discharge and single questions calculated from the 122 family members who completed the questionnaire. Percentages for the rehabilitation phase are calculated from the 113 family members who completed questions about the rehabilitation unit

^c^Parent experience of paediatric care (PEPC) scales: Ns = Nursing services, Ds = Doctor services, O = Organization, I = Information–examinations and tests, DI = Discharge information, HF = Hospital facilities, PEPC-S = single item in addition to the scales in the PEPC questionnaire. (−) = New items in the FECQ

^d^Item removed from the final scale in the PEPC-questionnaire

^e^Item removed from the final scale in the FECQ-TBI

^f^Scoring corresponds to scoring of other items: higher values represent better results

The original scoring method was preserved. Each item was scored from 1 (worst experience) to 5 (best experience). Items related to experiences with the provided health care ranged from 1-*not at all* to 5-*to a very large extent*. Negative items were re-coded before summation; thus, a higher score represents better experience. The questionnaire did not include an opportunity to respond “not applicable”.

### Data collection

The self-administered FECQ-TBI (version 1) was mailed to 171 family members of patients with severe TBI who were injured between 2009 and 2011 and who participated in a Norwegian multicentre study [31]. Data were collected from family members 3 and 12 months after injury for patients injured in 2010 and only 12 months after injury for other patients. In the family experiences of care study, data collected 12 months after injury were preferred.

### Statistics

The statistical analyses were conducted using the SPSS version 22.0. Items with more than 10% missing were excluded if also supported by consensus in the expert group (to avoid excluding conceptually important items). Consensus meant agreement among the authors AA, UM and CA. Items covering discharge and items regarding hospital facilities were treated as assessing distinct concepts. An exploratory factor analysis (EFA) was used to identify the number of underlying factor structures that adequately summarise the FECQ items. The principal component analysis (PCA) method was preferred in order to capture all variance. The component solution was promax rotated. Components with eigenvalues > 1 were retained, and loadings < 0.4 were suppressed. The item distribution indicated that most items were positively skewed and thus not normally distributed. The PCA was therefore based on Spearman’s rank-order correlation coefficients, which is a non-parametric method that is less vulnerable to skewed data. Separate PCAs were conducted on a) the 31 items with experiences relating to the acute and rehabilitation departments, b) the six items with experiences relating to discharge, and c) the seven items with experiences relating to hospital facilities (an overview of the items can be observed in Table 1). The overall single-item questions were not topic for factor analysis [12, 13, 22].

The internal consistency of the identified subscales was evaluated using the Cronbach’s alpha coefficient. Values > 0.70 were deemed satisfactory. Construct and criterion related validity was evaluated examining the structural relationships (i.e. correlation coefficients) between the subscale scores and between the subscale scores and the other included measures, respectively [26]. These associations were examined by Spearman’s rank correlations.

## Results

### Participants

A total of 171 close family members of patients with severe TBI were contacted by telephone or mail, and 122 completed the questionnaire (response rate 71%). Characteristics of non-participating family members were not available. Regarding patients’ characteristics, the proportion of male patients was higher in the participating group (90%) compared to non-participants (78%) (*p* < 0.05). There were no differences in patients’ age, marital status, educational level, acute injury severity or functional outcome measured with the Glasgow Outcome Scale Extended [32]. The family members were parents (43%), spouses/cohabitants (41%) or other relatives or close persons (16%). The questionnaire data 12 months post-injury were used (*n* = 117). We additionally included five family members who only responded at three months follow-up.

### Data quality and missing items

Table 1 provides descriptive statistics of all the FECQ-TBI items in the version-1-questionnaire (degree of missing items, means and standard deviations). The items asking for experiences with the acute and rehabilitation departments had small percentages of missing data (ranging from 1.6 to 5.3%), indicating that all items were endorsed. Two items regarding arrangements for the post-discharge period had substantially higher proportions of missing values (≥25%) and were therefore excluded (Table 1, Discharge period, items 5 and 6). Another two items related to hospital facilities for family members had high proportions of missing values (19.5 and 23%, respectively), but were retained for further analyses due to the lower number of items within this domain (Table 1, Hospital facilities, items 5 and 6).

### EFA on the acute and rehabilitation departments’ items

The PCA yielded four components, as shown in Table 2. Two items were excluded due to equally high side-loadings and low loadings, i.e., *“one doctor mainly responsible in the acute phase”* and *“rehabilitation: information about tests and examinations”*. A second PCA with these two items excluded again yielded four components with eigenvalues >1 (Table 3): the first component summarised items related to rehabilitation organisation, collaboration and competence; the second component summarised items related to the acute phase; the third component summarised items related to rehabilitation information and the involvement of family members; and the fourth component summarised three items related to rehabilitation organisation/staff stability. As the fourth component contained three items and was theoretically strongly related to the first component, also showing a strong correlation (*r* =0.59), a final PCA only extracting three components was conducted (Table 4). The items belonging to components 1 and 4 then combined in the first component now covering rehabilitation organization, cooperation and competence, while the second and the third components were the same as in Table 3.

**Table 2:** Initial factor analysis of *all the 31 items* related to the acute and rehabilitation phases

	Factor loadings^a^			
Item	*1:* *Rehabilitation* Organization, cooperation, competence	*2:* *Acute* Organization and Information	*3: Rehabilitation* Information and Involvement	*4: Rehabilitation* Organization – stability
Re: Felt assure regarding necessary care and rehabilitation	.92			
Re: Staff seemed professionally competent	.88			
Re: Staff committed themselves to patient	.83			
Re: Care and rehabilitation well planned	.83			
Re: Thoughtfulness, care for **the patient**	.81			
Re: Staff provided assistance with the patient	.75			
Re: Fixed group nurses	.69			
Re: Staff provided coordinated information	.67			
Re: Staff collaboration	.67			
Re: Adequate explanation about the purpose of the rehabilitative care	.50			
Ac: Thoughtfulness, care for **the patient**		.87		
Ac: Staff gave understandable information		.85		
Ac: Staff collaboration		.83		
Ac: Staff professionally competent		.82		
Ac: Fixed group nurses		.81		
Ac: Information tests and examinations		.80		
Ac: Care and rehabilitation well planned		.79		
Ac: Staff took account of family situation		.71	.45	
Ac: Thoughtfulness, care for **the relative**		.71	.49	
Ac: Staff interested in your opinions		.63	.55	
Re: Thoughtfulness, care for **the relative**			.80	
Re: Informed what you could contribute with			.78	
Re: Staff took account of family situation			.77	
Re: Staff interested in your opinions			.75	
Re: Information about rights (vocational opportunities, pensions, insurance, support)			.62	
Re: Staff gave understandable information	.45		.61	
*Re: Information tests and examinations* ^*b*^			*.38*	
Re: Had a fixed rehabilitation contact-person				.73
Re: One doctor mainly responsible				.71
*Ac: One doctor mainly responsible* ^*b*^		*.58*		*.59*
Re: Fixed group of other therapists				.53

*Ac* Items related to the acute phase (intensive care unit or a surgical department)

*Re* Items related to in-patient rehabilitation

^a^Factor loadings < 0.4 are suppressed

^*b*^Items in Italics was removed before the next factor analysis due to low or cross-loadings

**Table 3:** Factor analysis with Principal Component Analysis after removing two non-fitting items of *29 items* related to the acute and rehabilitation phase

	Factor loadings^a^			
Item	*1:* *Rehabilitation* Organization, cooperation, competence	*2:* *Acute* Organization and Information	*3: Rehabilitation* Information and Involvement	*4: Rehabilitation* Organization – stability
Re: Felt assure regarding necessary care and rehabilitation	.92			
Re: Staff seemed professionally competent	.92			
Re: Thoughtfulness, care for **the patient**	.85			
Re: Care and rehabilitation well planned	.82			
Re: Staff committed themselves to patient	.81			
Re: Staff provided assistance with the patient	.75			
Re: Staff provided coordinated information	.68			
Re: Staff collaboration	.64			
Re: Fixed group nurses	.61			
Re: Adequate explanation about the purpose of the rehabilitative care	.50			
Ac: Thoughtfulness, care for **the patient**		.87		
Ac: Staff collaboration		.86		
Ac: Staff gave understandable information		.84		
Ac: Fixed group nurses		.84		
Ac: Care and rehabilitation well planned		.82		
Ac: Staff seemed professionally competent		.82		
Ac: Information tests and examinations		.81		
Ac: Staff took account of family situation		.72	.44	
Ac: Thoughtfulness, care for **the relative**		.72	.48	
Ac: Staff interested in your opinions		.63	.54	
Re: Thoughtfulness, care for **the relative**			.79	
Re: Staff took account of family situation			.76	
Re: Informed what you could contribute with			.76	
Re: Staff interested in your opinions			.74	
Re: Informed about rights (vocational opportunities, pensions, insurance, support)			.60	.42
Re: Staff gave understandable information	.49		.60	
Re: Had a fixed rehabilitation contact-person				.86
Re: One doctor mainly responsible				.76
Re: Fixed group other therapists				.64
Cumulative % of Variance explained	49.47	64.77	71.09	75.20
Component correlation 1:				
2:	.39			
3:	.57	.45		
4:	.59	.19	.29	

*Ac* Items about the acute phase (intensive care unit or a surgical department)

*Re* Items about the inpatient rehabilitation phase

^a^Factor loadings < 0.4 are suppressed

**Table 4:** Factor analysis with Principal Component Analysis and a 3-factor solution with *29 items* assessing experiences from the acute and rehabilitation departments

	Factor loadings^a^		
Items	*1:* *Rehabilitation* Organization, cooperation, competence, stability	*2:* *Acute* Organization and Information	*3: Rehabilitation* Information and Involvement
Re: Fixed group nurses	.89		
Re: Care and rehabilitation well planned	.79		
Re: Staff collaboration	.78		
Re: Fixed group other therapists	.77		
Re: One doctor mainly responsible	.75		
Re: Staff committed themselves to patient	.74		
Re: Had a fixed rehabilitation contact-person	.74		
Re: Felt assure regarding necessary care and rehabilitation	.73		
Re: Staff provided coordinated information	.62		
Re: Staff seemed professionally competent	.61		
Re: Adequate explanation about the purpose of the rehabilitative care	.55		
Re: Staff provided assistance with the patient	.47		
Re: Thoughtfulness, care for **the patient**	.45		*.44* ^*b*^
Ac: Thoughtfulness, care for **the patient**		.88	
Ac: Staff collaboration		.86	
Ac: Staff gave understandable information		.85	
Ac: Fixed group nurses		.83	
Ac: Staff seemed professionally competent		.83	
Ac: Care and rehabilitation well planned		.82	
Ac: Information tests and examinations		.81	
Ac: Staff took account of family situation		.72	*.45* ^*b*^
Ac: Thoughtfulness, care for **the relative**		.71	*.46* ^*b*^
Ac: Staff interested in your opinions		.62	*.55* ^*b*^
Re: Staff took account of family situation			.87
Re: Thoughtfulness, care for **the relative**			.85
Re: Staff interested in your opinions			.79
Re: Informed what you could contribute with			.79
Re: Staff gave understandable information			.75
Re: Staff informed about rights (vocational opportunities, pensions, insurance, support)			.54
Cumulative % of Variance explained	49.47	64.77	71.09
Cronbach’s alpha	.95	.94	.93
Component correlation 1:			
2:	.33		
3:	.57	.49	

*Ac* Items assessing the acute phase (intensive care unit or a surgical department)

*Re* Items assessing the inpatient rehabilitation phase

^a^Factor loadings < 0.4 are suppressed

^*b*^Removed from the final scale

The items related to the acute phase can be divided into two subscales that correspond to rehabilitation items: acute- organisation, cooperation (5 items; Cronbach’s alpha 0.89) and acute- information and involvement (5 items; Cronbach’s alpha 0.94). This division gives us the ability to compare identical questions of experiences in the acute and rehabilitation departments.

### EFA on the discharge and on the hospital facilities items

The four items asking for experiences related to the discharge period were adequately summarised using a single component (eigenvalue = 2.82, *R* ^*2* =^ 70.4%). All items had high loadings (range 0.75–0.88), and the Cronbachs alpha was high (α = 0.86). The seven items asking for experiences with hospital facilities during rehabilitation were adequately summarised using a single component, however one item (i.e. *“available activities for the patient”*) was excluded due to a low loading. A new PCA with six items yielded two components (eigenvalues 3.46 and 1.22, *R* ^*2*^ = 78.0%): one patient component regarding patients’ experiences with cleaning, bathroom standards, the noise level and food (four items), and a second family member component assessing experiences with food and accommodations (two items). The factor loadings ranged from 0.68–0.95 and the Cronbach’s alpha coefficients were 0.86 and 0.80, respectively. The final developed questionnaire with scoring procedure is available as a Additional file 1. The acute stage item about “one doctor mainly responsible” was kept as a single item but is not included in any of the subscales.

### Indications of construct validity

The correlations between the subscale scores were all positive and statistically significant, as hypothesised (range 0.34–0.80), except a single non-significant correlation between hospital facilities-family and acute organisation and information (*r* = 0.16). See Additional file 2: Table S1. Experiences from the discharge period were moderately to strongly correlated with other scales (0.29–0.66). Overall satisfaction with health care was moderately to strongly significantly related to all subscales (range 0.27–0.57) except hospital facilities-family. Overall satisfaction with family care was significantly correlated to all subscales (correlation coefficients 0.29–0.63), with the strongest correlations to the acute subscales. As expected, the subscales were negatively correlated to incorrect treatment and problems with staff (range −0.12 to −0.45). Problems with transfer between departments were negatively correlated to the acute and rehabilitation subscales (−0.24 to −0.32) and were not significantly correlated to experiences in the discharge period. Patient age was not significantly correlated to any subscales. The tests supported the validity of the questionnaire.

## Discussion

In this multicentre study of family experiences of care after severe traumatic brain injury, a multidimensional scale was developed and evaluated. The scale was constructed based on a parent experience questionnaire [22] after a review of the literature and interviews with family members to investigate experiences with in-hospital care, treatment and rehabilitation. This is the first multidimensional constructed instrument designed for family members after TBI that measures both general satisfaction and experiences in six dimensions: acute care, rehabilitation-organisation, rehabilitation-information, discharge, hospital facilities- patients and hospital facilities-family. Good psychometric properties were found for the developed instrument.

General questions regarding treatment satisfaction are often given high ratings [11, 17, 21] and this questionnaire was designed to assess experiences with specific aspects of care that are more useful for quality improvement [16]. The importance of the assessment lies in the consistently positive associations among experiences with care and patient safety, effectiveness and health outcomes [10]. Furthermore, unmet family needs are regularly revealed when investigating the situation of family members to patients with TBI during in-hospital rehabilitation [4, 33]. Rotondi et al. (2007) found that families described their needs via phases that paralleled transitions in settings, i.e., acute care, in-patient rehabilitation, returns home and community setting [7]. The new questionnaire FECQ-TBI covers central phases related to in-hospital care.

Many patient and parent satisfaction and experiences questionnaires share common properties and contain the same or corresponding items [14]. To a lesser degree, this is also the case if instruments are not generic, but rather are developed for specific patient groups [13]. Of the domains of general importance for health care services, of great significance is *information*, an area often found in family satisfaction studies to require improvement [25, 34]. Additionally, instruments on family needs after TBI focus on health information together with support and involvement with care [33, 35]: these areas are included in the factor/subscale *information* in the FECQ-TBI. The second rehabilitation-related factor is *organization,* which is a significantly important domain for seriously ill patients in the intensive care unit (ICU) and for patients with a long length of stay [36]. Included in the organisation subscale is stability of personnel, in accordance with a qualitative study reporting that sustained connections with professionals are of utmost importance in rehabilitation [37]. *Hospital facilities*, such as availability and quality of accommodation for family members, was a theme that emerged. In a neonatal ICU a higher standard has been observed to relate to improvements of satisfaction and work quality for the parents [38]. Information regarding the long-term consequences of the injury and post-discharge arrangements for rehabilitation was emphasised, in accordance with previous findings [7]. Other items added from the focus group approach related to experiences of feeling safe regarding sufficient care and rehabilitation, the need for explanations regarding the purpose of rehabilitation, the level of staff engagement in the patient’s situation and the need for information regarding social security rights. In contrast to family satisfaction instruments designed for ICUs [39], decision-making was not a separate concept in this study. Because most questions added through the focus group and by expert group judgement were of a general character, the final FECQ-TBI could easily be applied to family members to other in-patient rehabilitation categories.

Although the psychometric properties, including construct validity, were good and were at least in line with comparable studies, this study had some limitations. First, the design of this study did not assess test-retest reliability. For now, we must rely on satisfactory findings from the PEPC questionnaire and modifications [22, 27]. Additionally, the focus group could have included a higher number of family members than three to secure a broader covering of themes. Our initial intention to organize a focus group with 4–5 participants failed of practical reasons. There is a lack of clear evidence based guidance about deciding on sample size, and the usual group size is between 4 and 12 participants [28]. However, the focus group was well prepared and based on a thorough literature search, and used the PEPC as a starting point to develop a structured interview guide. Caregivers varied in respect to strategically important aspects and rich discussions between participants revealed important care experiences. The intention of developing a questionnaire that covers most central care experiences was most probable achieved. Content adequacy and validity could be checked in further studies to assure the usefulness of the questionnaire in countries with different health care pathways and care delivery than Norway [35]. Another possible limitation is that not all suggested scales are based on factor analysis of all of the available items. In line with the practice of other instrument creators, scales can differ from factors and can be based on conceptual considerations when the proposed scales/subscales otherwise fulfil necessary psychometric properties [13, 22]. A further limitation is the number of items with high proportions of missing data. To avoid losing all information on the family members’ experiences with hospital facilities, we choose to keep two items despite their high levels of missing data. Although the proportion of missing data was high due to limited relevance for all family members, we concluded that the dimension was important for many families with patients experiencing long hospital stays [25, 38]. However, to further establish the questionnaires validity and utility use in other real-world samples would be preferable.

The strengths of this study are its multicentre national design and the relatively high questionnaire response rate of 71%, which is higher than in comparable studies (range 44–55%) [22, 27, 39, 40]. The higher response rate is explained by the fact that severe TBI patients and their family members have a closer connection to a hospital than do other patients and relatives participating in hospital surveys. Examination of differences between respondent and non-respondent caregivers was not possible as data on non-respondent caregivers was not available. However, patient-related characteristics were similar in the respondent and non-respondent group, except a small difference in the proportion of male patients, supporting representativeness.

## Conclusions

The FECQ-TBI includes important experiences of care related to in-hospital treatment phases. The questionnaire has high data quality, internal consistency and construct validity and can be used in hospitals with rehabilitation departments for quality improvement.

## Additional files

Additional file 1: The Family Experiences of in-hospital Care Questionnaire in severe Traumatic Brain Injury (FECQ-TBI) – final version. (PDF 75 kb)
Additional file 2: Table S1. Internal correlations among subscale scores and correlations with single- items questions. (DOCX 17 kb)
