# Supplementary material for: The family experiences of in-hospital care questionnaire in severe traumatic brain injury (FECQ-TBI): a validation study
Source: BMC Health Serv Res. 2016 Nov 28;16:675. doi: 10.1186/s12913-016-1884-6 (PMC5126854; doi:10.1186/s12913-016-1884-6)
Supplement: Additional file 1: — The Family Experiences of in-hospital Care Questionnaire in severe Traumatic Brain Injury (FECQ-TBI) – final version. (PDF 75 kb) [file 12913_2016_1884_MOESM1_ESM.pdf]

# THE FAMILY EXPERIENCES OF IN-HOSPITAL CARE QUESTIONNAIRE IN SEVERE TRAUMATIC BRAIN INJURY (FECQ-TBI)

**Please note: This form should only be filled in if the person who suffered the head injury has been discharged from their first period in hospital (including the rehabilitation unit, if relevant).**

*We would like to hear about your experience as a relative during the time in hospital. We are interested to know about the experience of close relatives during different phases of the patient's time in hospital. If the patient has been hospitalized several times, please answer these questions on the basis of their first period in hospital.*

| | | Very dis-satisfied | Dis-satisfied | Neither satisfied nor dis-satisfied | Satisfied | Very satisfied |
|---|---|---|---|---|---|---|
| 1 | Overall, how satisfied or dissatisfied are you with the care, treatment and rehabilitation the patient received at the hospital. *(Please take into account both the acute phase and the rehabilitation phase)* | ❑ | ❑ | ❑ | ❑ | ❑ |
| 2 | Overall, how satisfied or dissatisfied are you with the way you were treated as a relative? | ❑ | ❑ | ❑ | ❑ | ❑ |

***Your experiences from the first phase, i.e. the intensive care ward, surgical ward or another ward other than a rehabilitation unit:***

| | | Not at all | To a low extent | To a moderate extent | To a high extent | To a very large extent |
|---|---|---|---|---|---|---|
| 3 | To what extent did you experience that there was one doctor who had primary responsibility for the patient? | ❑ | ❑ | ❑ | ❑ | ❑ |
| 4 | To what extent did you experience that there was a fixed group of nurses who took care of you? | ❑ | ❑ | ❑ | ❑ | ❑ |
| 5 | To what extent did you experience that the staff collaborated about the treatment and care the patient received? | ❑ | ❑ | ❑ | ❑ | ❑ |
| 6 | Did you perceive that the treatment, care and rehabilitation the patient received during the acute phase followed a well thought-out plan? | ❑ | ❑ | ❑ | ❑ | ❑ |
| 7 | Did you receive the results of tests and examinations that you considered to be necessary during this phase? | ❑ | ❑ | ❑ | ❑ | ❑ |

| | **To what extent do you think the personnel:** (only one response per question) | Not at all | To a low extent | To a moderate extent | To a high extent | To a very large extent |
|---|---|---|---|---|---|---|
| 8 | Took your family situation into account? | ❑ | ❑ | ❑ | ❑ | ❑ |
| 9 | Demonstrated thoughtfulness and care for *the patient*? | ❑ | ❑ | ❑ | ❑ | ❑ |
| 10 | Demonstrated thoughtfulness and care for *you as a relative*? | ❑ | ❑ | ❑ | ❑ | ❑ |
| 11 | Were interested in hearing your opinions as a relative? | ❑ | ❑ | ❑ | ❑ | ❑ |
| 12 | Gave you explanations and information that you understood? | ❑ | ❑ | ❑ | ❑ | ❑ |
| 13 | Seemed professionally competent? | ❑ | ❑ | ❑ | ❑ | ❑ |

**Complete this section if the patient was admitted to a rehabilitation ward.**

## *What are your experiences of the rehabilitation ward / unit?*

| | | Not at all | To a low extent | To a moderate extent | To a high extent | To a very large extent |
|---|---|---|---|---|---|---|
| 14 | To what extent did you experience that there was one doctor who had primary care of the patient? | ❑ | ❑ | ❑ | ❑ | ❑ |
| 15 | To what extent did you experience that there was a fixed group of nurses who took care of you? | ❑ | ❑ | ❑ | ❑ | ❑ |
| 16 | To what extent did you experience that there was a fixed group of other therapists (e.g. physiotherapist, occupational therapist, social worker and speech therapist) who took care of you? | ❑ | ❑ | ❑ | ❑ | ❑ |
| 17 | To what extent did you experience that the staff collaborated about the treatment and care the patient received? | ❑ | ❑ | ❑ | ❑ | ❑ |
| 18 | Did you perceive that the treatment, care and rehabilitation the patient received during the rehabilitation phase followed a well thought-out plan? | ❑ | ❑ | ❑ | ❑ | ❑ |
| 19 | To what extent did you feel assured that the patient received the necessary treatment, care and rehabilitation? | ❑ | ❑ | ❑ | ❑ | ❑ |
| 20 | To what extent was the purpose of the rehabilitative care the patient received explained to you? | ❑ | ❑ | ❑ | ❑ | ❑ |
| 21 | Did you perceive that the staff committed themselves to the patient's circumstances? | ❑ | ❑ | ❑ | ❑ | ❑ |
| 22 | Did you experience that you had a fixed contact at the rehabilitation ward? | ❑ | ❑ | ❑ | ❑ | ❑ |
| 23 | To what extent did you receive information about the patient's rights (e.g. vocational measures, social security benefits, insurance and social services)? | ❑ | ❑ | ❑ | ❑ | ❑ |

| | **To what extent do you think the personnel:** (only one response per question) | Not at all | To a low extent | To a moderate extent | To a high extent | To a very large extent |
|---|---|---|---|---|---|---|
| 24 | Took your family situation into account? | ❑ | ❑ | ❑ | ❑ | ❑ |
| 25 | Provided assistance / help with the patient during the stay? | ❑ | ❑ | ❑ | ❑ | ❑ |
| 26 | Informed you about what you as a relative could contribute with at the hospital? | ❑ | ❑ | ❑ | ❑ | ❑ |
| 27 | Demonstrated thoughtfulness and care for *the patient*? | ❑ | ❑ | ❑ | ❑ | ❑ |
| 28 | Demonstrated thoughtfulness and care for *you as a relative*? | ❑ | ❑ | ❑ | ❑ | ❑ |
| 29 | Were interested in hearing your opinions as a relative? | ❑ | ❑ | ❑ | ❑ | ❑ |
| 30 | Gave you explanations and information that you understood? | ❑ | ❑ | ❑ | ❑ | ❑ |
| 31 | Seemed professionally competent? | ❑ | ❑ | ❑ | ❑ | ❑ |
| 32 | Provided coordinated information about the patient? | ❑ | ❑ | ❑ | ❑ | ❑ |

## What did you think about the following conditions in the rehabilitation ward?

| | | Very poor | Moder-ately poor | Mixed | Moder-ately good | Very good |
|---|---|---|---|---|---|---|
| 33 | Cleanliness | ❑ | ❑ | ❑ | ❑ | ❑ |
| 34 | Bathroom/shower/toilet facilities | ❑ | ❑ | ❑ | ❑ | ❑ |
| 35 | Peace and quiet in the patient's room | ❑ | ❑ | ❑ | ❑ | ❑ |
| 36 | Meals for the patient | ❑ | ❑ | ❑ | ❑ | ❑ |
| 37 | Meals for the relative(s) | ❑ | ❑ | ❑ | ❑ | ❑ |
| 38 | Rest room and accommodation provisions for the relative(s) | ❑ | ❑ | ❑ | ❑ | ❑ |

| | | Yes | Partly | No |
|---|---|---|---|---|
| 39 | Did the patient have his/her own room? | ❑ | ❑ | ❑ |

## *The questions below deal with the time before the patient's discharge*

| | | Not at all | To a low extent | To a moderate extent | To a high extent | To a very large extent |
|---|---|---|---|---|---|---|
| 40 | Did you receive the information you considered to be necessary about the period after discharge from hospital? | ❑ | ❑ | ❑ | ❑ | ❑ |
| 41 | At the time of returning home, were you confident that you could manage the necessary follow-up of the patient? | ❑ | ❑ | ❑ | ❑ | ❑ |
| 42 | Did you receive information about what you could do in the event of problems or unexpected events after your return home? | ❑ | ❑ | ❑ | ❑ | ❑ |
| 43 | To what extent did you receive information about the short and long term consequences of head injuries? | ❑ | ❑ | ❑ | ❑ | ❑ |

## *The questions below deal with the time in hospital as a whole*

| | | Not at all | To a low extent | To a moderate extent | To a high extent | To a very large extent |
|---|---|---|---|---|---|---|
| 44 | Do you believe that the patient in any way received the wrong treatment (based on what you are able to judge)? | ❑ | ❑ | ❑ | ❑ | ❑ |
| 45 | Were you angry, distressed or disappointed with the staff during the time at hospital? | ❑ | ❑ | ❑ | ❑ | ❑ |
| 46 | Did you experience any adverse incidents in connection with the patient's transfer from one ward to another or from one hospital to another? | ❑ | ❑ | ❑ | ❑ | ❑ |
| 47 | Were your financial needs taken care of? | ❑ | ❑ | ❑ | ❑ | ❑ |
| 48 | Were children involved? ❑ Yes ❑ No | | | | | |
| 49 | If yes, were the children taken care of? | ❑ | ❑ | ❑ | ❑ | ❑ |

# SCORING PROCEDURE OF THE FAMILY EXPERIENCES OF IN-HOSPITAL CARE QUESTIONNAIRE IN SEVERE TRAUMATIC BRAIN INJURY (FECQ-TBI)

The FECQ-TBI was developed in 2015 to assess important aspects of in-hospital care in the acute and rehabilitation phases from a family perspective. The FECQ-TBI was designed to record the quality of care experiences separately for different phases of care to be able to make relevant comparisons.

**All items are scored from 1 (worst experience) to 5 (best experience), except for items 44-46 where 1 is the best experience and 5 the worst experience.**

- The items 1 and 2 assess your overall experience and are scored: very dissatisfied =1, dissatisfied =2, neither satisfied nor dissatisfied = 3, satisfied = 4 and very satisfied =5.
- Items 3-32, 40-47 and 49, are scored as: not at all =1, to a low extent =2, to a moderate extent =3, to a high extent =4 and to a very large extent =5.
- Items 33-38 are scored as: very poor =1, moderately poor = 2, mixed = 3, moderately good =4 and very good = 5.

**Scoring procedure of the FECQ-TBI**

- Acute organization and information subscale = mean of items 4, 5, 6, 7, 8, 9, 10, 11, 12 and 13.
- Rehabilitation organization subscale= mean of items 14, 15, 16, 17, 18, 19, 20, 21, 22, 25, 27, 31 and 32.
- Rehabilitation information subscale = mean of items 23, 24, 26, 28, 29 and 30.
- Discharge period subscale = mean of items 40, 41, 42 and 43.
- Hospital facilities patient subscale= mean of items 33, 34, 35 and 36.
- Hospital facilities family member subscale = mean of items 37 and 38.
- For comparison between identical questions in the acute versus the rehabilitation phase, the following items are identical (acute/rehabilitation): 3/14, 4/15, 5/17, 6/18, 8/24, 9/27, 10/28, 11/29, 12/30 and 13/31.

***References:***

*Anke A, Manskow US, Friborg O, Røe C, Arntzen C: The Family Experiences of in-hospital Care Questionnaire in Severe Traumatic Brain Injury (FECQ-TBI): A validation study. BMC Health Services Research. 2016.*

*Garratt AM, Bjertnaes OA, Barlinn J: Parent experiences of paediatric care (PEPC) questionnaire: reliability and validity following a national survey. Acta paediatrica. 2007; 96(2):246-252.*
