# Supplementary material for: The family experiences of in-hospital care questionnaire in severe traumatic brain injury (FECQ-TBI): a validation study
Source: BMC Health Serv Res. 2016 Nov 28;16:675. doi: 10.1186/s12913-016-1884-6 (PMC5126854; doi:10.1186/s12913-016-1884-6)
Supplement: Additional file 2: Table S1. — Internal correlations among subscale scores and correlations with single- items questions. (DOCX 17 kb) [file 12913_2016_1884_MOESM2_ESM.docx]

Additional file 2: Table S5. Internal correlations^1^ among subscale scores and correlations^1^ with single- items questions.

| **Scale**/item | Acute-  Organ-5 | Acute-Inform-5 | Rehab-Organ-13 | Rehab-Inform-6 | Hospital facilities-patient-4 | Hospital facilities-family-2 | Discharge-4 |
| --- | --- | --- | --- | --- | --- | --- | --- |
| **Acute-Information** | .74^§^ |  |  |  |  |  |  |
| **Rehab-Organization** | .45^§^ | .42^§^ |  |  |  |  |  |
| **Rehab-Information** | .34^§^ | .54^§^ | .80^§^ |  |  |  |  |
| **Hospital facilities-patient** | .40^§^ | .36^§^ | .55^§^ | .49^§^ |  |  |  |
| **Hospital facilities-family** | .16 | .16 | .34^§^ | .36^§^ | .40^§^ |  |  |
| **Discharge** | .29^§^ | .51^§^ | .55^§^ | .66^§^ | .41^§^ | .31^#^ |  |
| Overall satisfaction care, treatment, rehab | .57^§^ | .52^§^ | .42^§^ | .33^§^ | .27^#^ | .19 | .42^§^ |
| Overall satisfaction – family care | .54^§^ | .63^§^ | .44^§^ | .43^§^ | .29^#^ | .28^#^ | .54^§^ |
| Extent of any incorrect treatment | -.34^§^ | -.36^§^ | -.40^§^ | -.33^§^ | -.21^$^ | -.12 | -.20^$^ |
| Extent of any problems with staff | -.43^§^ | -.45^§^ | -.33^§^ | -.28^#^ | -.20^$^ | -.22^$^ | -.24^$^ |
| Problems transfer between departments | -.29^#^ | -.32^#^ | -.24^$^ | -.25^#^ | -.09 | .01 | -.15 |
| Patients age | -.08 | -.13 | -.03 | -.12 | .04 | -.04 | -.13 |

N=122 in Acute- Organization and Acute-Information scales. N=111/112 in Rehab-Organization, Rehab-Information, Hospital facilities, Discharge subscales.

^1^ Spearman´s rank correlations

^§^ <0.001, ^#^ <0.01, ^$^ <0.05
